# Westminster Hospital

**Published:** 1829-10

**Authors:** 


### HOSPITAL REPORTS.

### WESTMINSTER HOSPITAL.

### *Operation for Impermeable Stricture. Death of the Patient.*

RICHARD REYNOLDS, æt. fifty-six, had suffered nearly thirty years from stricture of the urethra. He was admitted April 15th, 1829. He passes upwards of a pint of urine, by drops, in the twenty-four hours. Bowels regular. General health but little impaired. A bougie cannot be passed, and each attempt to introduce the instrument has given rise to severe rigor. The seat of the stricture is supposed to be at the bulb of the urethra.

July 25.—Mr. WHITE determined to make an incision in the perineum, and cut through the stricture, which there was no hope of curing by common means. The patient was placed as for the operation of lithotomy. A straight staff was passed down to the stricture, and an incision made, an inch long, exactly in the raphé downwards to within half an inch of the anus. The index finger of the left hand was now introduced into the wound; the bulb of the urethra was raised; the membranous portion of the urethra opened, and the stricture divided. Some difficulty was experienced in passing a catheter into the bladder, which is by no means uncommon in this operation. Mr. White did not succeed in his attempts to pass the instrument; after which, Mr. GUTHRIE, having changed the posture of the patient, succeeded in introducing a female catheter. It was fixed in the urethra by a roller, and a T bandage was applied. Some hours after the operation, the man passed a pint of urine through the catheter.

26th.—Has had an uncomfortable restless night. Tongue foul, pulse eighty-five, skin hot; bowels open; urine has passed freely by the catheter.

28th.—Continues feverish. Pain in the perineum; urine high coloured and thick. Ordered saline medicine.

From this time to August 12th, the symptoms of general irritation and fever continued to increase. On the 12th the wound had a sloughy appearance, and the urine passed as freely through it as through the catheter. On the 16th he died.

*Examination twenty-four hours post mortem.*—The bladder was found to be much thickened. The ureters were enlarged, and the canal of the urethra was obliterated; on each side there was a false passage. There was neither perineal fistula nor infiltration of urine in the cellular membrane.

